# Extramedullary paraspinal hematopoiesis in hereditary spherocytosis

**DOI:** 10.4103/1817-1737.39640

**Published:** 2008

**Authors:** P. Gogia, R. Goel, S. Nayar

**Affiliations:** *Department of Pulmonology, Jaipur Golden Hospital, New Delhi, India*

**Keywords:** Extramedullary hematopoiesis, hereditary spherocytosis, posterior mediastinal masses

## Abstract

Hereditary spherocytosis (HS) is a common inherited hemolytic anemia due to red cell membrane defects. Extramedullary hematopoiesis is a compensatory response to insufficient bone marrow blood cell production. The preferred sites of extramedullary hematopoietic involvement are the spleen, liver and lymph nodes; but in HS, the posterior paravertebral mediastinum is also commonly involved. We report a case of a 50-year-old male who presented to us in respiratory distress and with bilateral paravertebral posterior mediastinal masses, which on trucut biopsy were found to be extra-hematopoietic masses; and the patient was found to have hereditary spherocytosis.

## Introduction

Extramedullary hematopoiesis may complicate chronic anemia states. Abnormal hematopoietic tissue usually develops in sites involved in hematopoiesis during fetal development, such as spleen, liver and kidneys; however, other locations, such as paraspinal tissue, especially in the posterior mediastinum, may also be involved.[1] Thoracic extramedullary hematopoiesis (EMH) associated with hereditary spherocytosis is a rare phenomenon.[2]

## Case History

A 50-year-old male presented to us with history of moderate-grade fever with respiratory distress for the last 4 days. He also gave history of anorexia for the last 15 days. He had a history of blunt trauma to the chest wall 1 month prior to the present problem. There was no history of cough, expectoration or any bowel and bladder complaints. He was initially evaluated for this at a different hospital. According to the records available, he was found to have a mass lesion on chest X ray. A contrast-enhanced computed tomography(CT) thorax done revealed a posterior mediastinal mass. A CT-guided FNAC was done from the lesion, and it was inconclusive; therefore, an open-lung biopsy was advised. The patient refused for the same and got admitted to our hospital. On admission, he was cachectic and toxic looking. He was febrile (temperature: 38.5°C). His pulse was 138/min, respiratory rate was 26/min and BP was 130/80 mmHg. He had pallor and icterus but there was no cyanosis, clubbing or lymphadenopathy. Chest examination revealed decreased breath sounds in the right interscapular area. His cardiovascular system examination was normal, while abdominal examination revealed a splenomegaly of 4 cm below left costal margin. The patient was admitted to the intensive care unit and was started on antibiotics and other supportive treatment. On investigation, he was found to have hemoglobin of 9.8 gm% (N: 13–17 g%); total leukocyte count - 23,000/cumm (N: 4–11,000/cumm) with a differential count of polymorphs 91%, lymphocytes 8%, eosinophils 1%, S. bilirubin (total) - 10.21 mg/dl (N: 0.2–1.0 mg/dl) (direct - 2.5 mg/dl), total protein 5.3 g/dl (N: 6.5–8.5 gm/dl), Alb - 2.7 mg/dl (N: 3.5–4.5 g%), SGOT - 22 IU/L; SGPT - 20 IU/L, alkaline phosphatase - 143 IU/L, sodium 135 mEq/L; potassium 3.6 mEq/L, BUN 11 mg/dl; S. creatinine 0.4 mg/dl, blood glucose 95 mg/dl. Coomb's tests - both direct and indirect - were negative.

**Figure 1:**
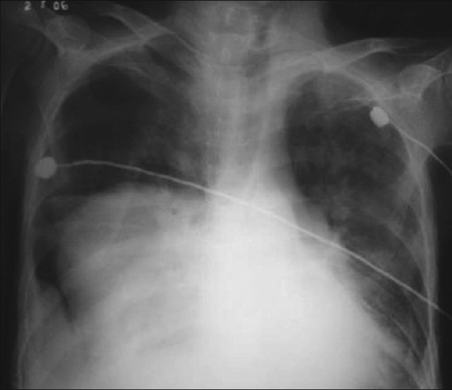
Chest X ray showing a bilateral mass lesions

**Figure 2:**
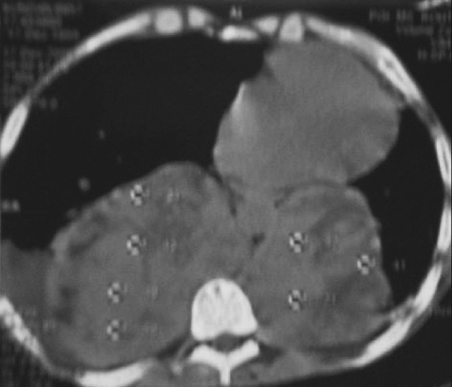
CECT thorax showing hypodense mass, seen in the posterior mediastinum involving bilateral paravertebral region extending from carina to diaphragm with right-sided pneumothorax with collapse of underlying lung

**Figure 3:**
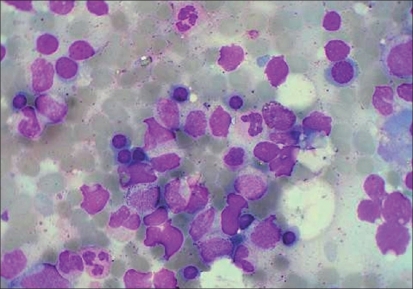
CT-guided biopsy from lung lesion showing hyperplastic erythroid bone marrow

Chest X ray showed a bilateral mass lesion [Figure 1]. CECT thorax showed hypodense mass, seen in the posterior mediastinum involving bilateral paravertebral region extending from carina to diaphragm with right-sided pneumothorax with collapse of underlying lung; and the radiologist's impression was a neurogenic tumor [Figure 2]. Ultrasound abdomen showed moderate splenomegaly. As the patient was too sick for an open-lung biopsy, a CT-guided trucut biopsy was done. The biopsy revealed hyperplastic erythroid bone marrow [Figure 3]. Repeat hematological and biochemical parameters showed an Hb 7.7 gm%, PCV 22.8%, TLC 14,300/cumm, differential count of polymorphs 93, lymphocytes 06, monocytes 1, platelets 90,000/cumm, reticulocyte count 7.0%, ESR 25 mm in the first hour. Peripheral smear showed red blood cells with moderate anisocytosis and comprised of normocytic normochromic cells, spherocytes and a fair number of polymorphic RBCs. Leukocyte counts were increased, and smear showed polymorphic leucocytosis. Platelets were moderately low. No immature cells or hemoparasite was seen. Osmotic fragility test showed markedly increased osmotic fragility of the red blood cells, and thereby a final diagnosis of hereditary spherocytosis with extramedullary hematopoiesis presenting as posterior mediastinal mass was made. The patient was given supportive treatment in the form of blood transfusion and antibiotics, but he could not be saved and succumbed due to overwhelming sepsis.

## Discussion

Hereditary spherocytosis (HS) is a common inherited hemolytic anemia due to red cell membrane defects. Extramedullary hematopoiesis is a physiological compensatory mechanism that occurs when the bone marrow is unable to maintain sufficient red cell production to supply body demand. Extramedullary hematopoiesis is a rare condition, characterized by the appearance of hematopoietic elements outside the bone marrow. It occurs primarily in patients with chronic myeloproliferative disorder or congenital hemolytic anemia.[3] The preferred sites of extramedullary hematopoietic involvement are the spleen, liver and lymph nodes; but in hereditary spherocytosis, the posterior paravertebral mediastinum is also commonly involved.[4] Fine-needle aspiration biopsy (FNAB) has been documented as a useful tool in diagnosing EMH tumor.[5] Previous case reports show that a majority of time diagnoses were made either at autopsy or on surgery.[2] In these cases, the chest X ray was characterized by rounded or lobulated masses of soft tissue density in posterior mediastinum. CT scan findings is characterized by homogenous round or lobulated soft-tissue masses, usually in the posterior mediastinum.[2] Thus, this case highlights the fact that intrathoracic extramedullary hematopoiesis is a rare disease and should be kept in the differential diagnosis of mediastinal masses, especially in patients with anemia and hemolytic jaundice. A good trucut biopsy can give us a correct diagnosis and thus can help to avoid an operation.

On MRI examination, extramedullary hematopoietic masses are seen as iso-intense lesions on both T1- and T2-weighted images. After administration of a paramagnetic agent, an intermediate enhancement of the masses is evident. The vertebral bodies have low-to-intermediate signal intensity as a result of displacement of fatty marrow by hematopoietic marrow, thereby highlighting the role of MRI in diagnosis of extramedullary hematopoietic masses.[6] MR-guided radiation therapy is a very effective treatment but only in cases where there is acute spinal cord compression which may lead to neurological complications.[7] Patients with asymptomatic masses should not be treated.
